# Socioeconomic Factors Associated with Knowledge on Tuberculosis among Adults in Ethiopia

**DOI:** 10.1155/2016/6207457

**Published:** 2016-02-01

**Authors:** Sifrash Meseret Gelaw

**Affiliations:** ^1^Unit 12 A, Tuscany Condo, Ayala Avenue 6751, Makati City, 1226 Metro Manila, Philippines; ^2^24th Floor Citibank Tower, Paseo De Roxas 8741, Makati City, 1226 Metro Manila, Philippines

## Abstract

*Background*. Ethiopia is among highly tuberculosis affected countries. This might be related to low level of awareness on the disease in the population. The objective of the study was to determine the level of tuberculosis knowledge and socioeconomic factors associated with it.* Methods*. The 2011 Ethiopia health and demographic survey data were used. Overall tuberculosis knowledge score was computed to evaluate the outcome variable. Multivariable logistic regression was employed to identify independent socioeconomic factors associated with low tuberculosis knowledge.* Results*. The overall tuberculosis knowledge was low, 44.05% (95% CI: 42.05–46.24%) among women and 32.3% (95% CI: 30.34–34.32%) among men. Rural women (AOR = 1.22) and youth, no formal education (women: AOR = 3.28, men: AOR = 7.42), attending only primary education (women: AOR = 1.95, men: AOR = 3.49), lowest wealth quintiles (women: AOR = 1.4, Men: AOR = 1.28), unskilled female manual workers (AOR = 4.15), female agricultural employee (AOR = 2.28), and lack of access to media (women: AOR = 1.52, men: AOR = 1.71) are significantly associated with low tuberculosis knowledge.* Conclusion*. The level of tuberculosis knowledge among adults in Ethiopia is low and varied by socioeconomic groups. Tuberculosis control programs should consider appropriate strategies for tuberculosis education, promotion, communication, and social mobilization to address the rural women, youths, the poor, less educated people, and unskilled workers.

## 1. Introduction

Tuberculosis (TB) is a widely recognized high-burden infectious disease in the world. It is a bacterial infection caused by a member* Mycobacterium* TB complex, commonly* Mycobacterium tuberculosis*. TB is transmitted by respiratory route when a patient is coughing or sneezing, and one strain of TB,* Mycobacterium bovis*, can be caused by drinking unboiled milk [1]. Risk of TB is high among population living in poverty, low socioeconomic groups, low income, immune-suppressed (including AIDS), and extreme age (old age and children) groups, certain ethnicity, migrants, and those exposed to animals (*Mycobacterium bovis*) [1, 2]. The risk of TB transmission also increases with the intimacy and duration of contact to TB patients and infectiousness of the TB patients [1].

Ethiopia is the second populous country in Africa with a population size of 88 million (July 2014 projection). Only 16% of the population lives in urban areas and the literacy rate is 42.7% [3, 4]. The country is one of the tuberculosis high-burden countries, standing on rank 8 under the 22 high TB burden countries in the world with an estimated prevalence and incidence rate of 394 and 261 cases per 100,000 inhabitants, respectively [5, 6]. The country begun to implement DOTS strategy in 1992. However, only approximately 60 to 70% of the population has access to DOTS services by 2005 [7, 8]. One of the major problems in controlling the TB burden is the poor awareness and knowledge of the population on TB [9, 10].

Low level of knowledge on TB can lead to complications and worse health outcomes increasing the transmission and delaying health seeking behavior, lack of adherence, resulting in multidrug resistance, treatment failure, and disease complication and death [11–13].

TB control could be significantly improved if more consideration was given to the population's knowledge and attitudes about TB and related health care-seeking behavior by directing efforts towards making individuals more informed and aware of all aspects of TB [14–16]. Advocating for the health of patients and developing policy to increase health literacy among the general public as well as health care professionals will create a supportive environment where optimal TB-related outcomes can be achieved [12, 17–20].

Different studies from different countries show the significance of the problem of low TB knowledge and the different socioeconomic factors associated with low TB knowledge in the different study populations. But on the other hand most studies done on TB knowledge in Ethiopia [9, 10, 17–22] as well as studies in different other high TB burden countries [23–29] are done among restricted population subgroups and are mainly health facility based studies which may overestimate the magnitude of the level of knowledge on TB and may not reflect the actual situation in the general population. Moreover, the TB knowledge components considered in these studies were not comprehensive items. These demonstrated the need for exploring large scale comprehensive community level study to recognize the different factors associated with knowledge on TB with the context of Ethiopian culture and socioeconomic status. Further detail analysis of large scale data will help to understand the level of knowledge on TB and associated factors at the country level, which will further guide to design evidence based country appropriate policy, strategy, intervention, service provision, and advocacy to enhance the TB control program. The aim of this study is to identify level of knowledge and socioeconomic factors associated with TB awareness and knowledge about TB transmission, TB symptoms, and TB treatment among adult population of Ethiopia.

## 2. Methods

To address the objectives of the study, secondary analysis of the 2011 Ethiopia Demographic and Health Survey (EDHS) was employed. The survey has used a cross-sectional study design. The survey interviewed a nationally representative sample population in 18,500 households involving 16,515 women aged 15–49 and 14,110 men aged 15–59 from all selected households. A stratified, two-stage cluster sampling method was used. Stratification was achieved by separating each region into urban and rural areas. In the first stage, 624 Enumeration Areas (EAs) were selected with probability proportional to the EA size and with independent selection in each sampling stratum. In the second stage, a fixed number of 30 households were selected for each EA. The survey used three questionnaires: the Household Questionnaire, the Woman's Questionnaire, and the Man's Questionnaire. The questionnaires were pretested, and consent was taken. Interviewers and supervisors were properly trained. A quality control team regularly visited the fieldwork to closely supervise and monitor field activities [3].

Due to the nonproportional allocation of the sample to the different regions and to their urban and rural areas, sampling weights are required for any analysis using 2011 EDHS data to ensure representativeness of the survey results at the national and regional level. Since the 2011 EDHS sample is a two-stage stratified cluster sample, sampling weights were calculated based on sampling probabilities separately for each sampling stage and for each cluster [3].

### 2.1. Data Management

Information on TB knowledge and socioeconomic factors was extracted from the large dataset. The different datasets for women and men were explored, variables relevant for the current study were identified, and the variables between the women and men dataset were harmonized. To have comparable age distribution between women and men, those with only 15–49 years were included in the analysis. Then the two datasets were matched to produce a subset of the original variables. The extracted data became the working dataset to address the objectives of the project.

### 2.2. Data Preparation

STATA version 12 was used to analyse the data. Exploration of the variables was made. Data were checked for errors by summarizing and producing frequencies and cross tabulations. Missing values were checked, identified, decoded, and excluded from the analysis.

### 2.3. Data Analysis Method

#### 2.3.1. Descriptive Analysis

Background characteristics of respondents were summarized and presented in tables. Absolute numbers and percentages are displayed. The proportion of respondents who are aware of TB and who have correct knowledge on TB transmission, TB symptoms, and TB treatment was calculated.

#### 2.3.2. Outcome Measure

The outcome variable, low comprehensive TB knowledge was measured as a composite indicator by considering responses related to TB awareness, modes of transmission, sign and symptoms, and treatment. Questions correctly answered were coded as “1” and incorrect answers as “0” during analysis. The lower terciles of the overall summary score were taken as low TB knowledge. The methodological approach in this study is based on different previous similar studies [9, 15]. In this study, the level of comprehensive TB knowledge was measured as the main outcome of interest. All the components of TB knowledge such as knowledge on TB transmissions, symptoms, and treatment are included as all the components are relevant with regard to their contribution to TB prevention and control as described above. Moreover, using an overall composite score allows straight forward comparison of TB knowledge among its different determinant factors. Since there was no study found on psychometric properties of DHS TB knowledge items, factor analysis was employed to examine the psychometric properties of the overall knowledge score.

#### 2.3.3. Factor Analysis and Measuring the Reliability of the Summary Scores

The factor structure of the different knowledge question items was investigated using Principal Components Analysis (PCA), to get clearance on the different dimension of the overall score index. A total of 15 items regarding assessing TB knowledge were included in the PCA. Factors with eigenvalue >1 were retained. Accordingly, five factors were identified and then rotation was done. Three variables with rotated loading factor less than 0.4 were omitted (these are transmission through sharing utensils, transmission through touching a person with TB, and the fact that tuberculosis can be cured). One variable which was loading on a factor but that was not interpretable was omitted (transmission through exposure to cold). In addition, an initial Cronbach Alpha was calculated (for the whole summary score) and for each variable an alternative alpha if a variable was sorted out was computed. One variable was omitted because the alternative alpha is higher than the initial alpha (transmission through sexual contact). A total of five variables were excluded. Cronbach's Alpha was calculated for checking consistency of the variables and found to be 0.62. This value is because an index of ten variables is quite small and alpha is dependent on the number of variables in the index. The value of alpha increased to 0.7 as the number of variables increased to fifteen. An overall summary score was calculated using raw values of the remaining variables with rotated factor loading greater than 0.4 on at least one interpretable factor. The lower terciles of the summary score were taken as the outcome measure (low TB knowledge).

From the above factor analysis result, four interpretable factors remained for the overall index construction. These factors are interpreted and qualitatively described as follows. The first component with three items is interpreted as “cough related symptoms and transmissions.” The second component with two items is interpreted as “food related symptoms and transmissions.” The third component with three items is interpreted as “key symptoms and transmissions.” The last component with two items is interpreted as “misconceptions.”

#### 2.3.4. Independent Variables

The following different socioeconomic factors are taken as independent variables in the analysis in this study: gender categorized as female and male; age in ten-year intervals; regions categorized into accessible regions (Tigray, Amhara, Oromiya, and SNNPR), hard to reach regions (Afar, Somali, Gambela, and Benishangul Gumuz), and city administrations and regions (Addis Ababa, Dire Dawa, and Harari); residence classified as urban and rural; wealth index (which was already calculated in the primary study based on a standard set of household assets, dwelling characteristics, and ownership of consumer items) categorized in to five where the first quintile was the poorest 20% of the households and fifth quintile the wealthiest 20% of the households; marital status classified as never in union, married, living with partner, widowed, divorced, and no longer living together/separated; education categorized into no education, primary, secondary, and more than secondary; occupational status classified as not working, professional/technical/managerial, clerical, sales and services, agricultural employee, skilled manual, and unskilled manual; frequency of exposure to mass media classified as reading newspaper, watching television, and listening radio; and exposure to community conversation program as yes and no response [3].

#### 2.3.5. Assessing Magnitude of Low TB Knowledge

Proportion of adults with low comprehensive TB knowledge was determined along with the corresponding 95% confidence interval (95% CI) at national and regional level as well as for urban and rural areas. The outcome measure, low comprehensive TB knowledge, was tabulated against socioeconomic factors. All analysis was stratified by sex, since there may be multiple interactions between sex and other independent variables.

#### 2.3.6. Bivariate and Multivariate Analysis of Association

Crude odds ratios (OR) along with the corresponding 95% confidence intervals were estimated using Mantel-Haenszel method to assess the association between the outcome of interest and each exposure factor. STATA SVY prefix command and weights were used to account for the complex sampling nature of the survey.

Binary logistic regression was employed to further analyse the data. The variables for subsequent analysis in the logistic regression model were selected based on prior information from the literature and/or statistical plausibility (*p* < 0.05 in the bivariate analysis). Forward logistic regression strategy was used for building the multivariate logistic regression model step by step.

In addition, interaction terms for effect modifiers were checked. Multicollinearity between independent variables was resolved by computing the collinearity matrix index. The likelihood ratios and the pseudo-*R* ^2^ in the STATA output of the final fit of the multivariable logistic regression model were checked and recorded to see the improvement of the model due to the independent variables included and the estimation of proportion of variability explained by the model. Crude and adjusted odds ratios along with their 95% confidence interval were presented. Associations were declared significant, if *p* < 0.05.

#### 2.3.7. Ethical Considerations

The project was approved by the Ethics Committee of LSHTM. The data source is a secondary human dataset from EDHS 2011. The primary EDHS 2011 has received clearance from the responsible bodies in Ethiopia. MEASRE DHS has given permission to further analyse the data and use it for the current study. There is no personal identifier attached to the dataset.

## 3. Results

### 3.1. Socioeconomic Characteristics

There were a total of 30,625 study participants in the dataset, of which 16,515 (53.9%) were women and 14,110 (46.1%) were men.

Majority, 68.8%, were from rural area and about half, 49.3%, were from the accessible regions (Tigray, Amhara, Oromia, and SNNPR). The age ranged from 15 to 49 years and 40.9% were 15 to 24 years of age. More than half (55.6%) were married and 32.9% have never been in union. About 41.6% of the participants had no formal education and 40.9% had only primary education. Almost one-third of the participants had no work and 38.3% were agricultural employees. About one in four (26.4%) had no access to newspaper, television, or radio. More than half (58.7%) of the participants have never heard of community conversions on various topics (Table 1).

### 3.2. Awareness and Correct Knowledge about TB

Awareness on TB is high, with 15,045 (89.9%) of women and 13,518 (95.1%) of men and overall 28,563 (92.3%) mentioning they had “heard of an illness called tuberculosis (TB).” But only 49.97% of women and 61.84% of men knew that TB is transmitted “by air when coughing or sneezing.” There were misconceptions of TB transmission; the common misconception was TB transmission by “sharing utensils” and by “exposure to cold,” with overall correct responses rate of 68.71% and 79.6%, respectively. Surprisingly, only 1.82% of females and 2.55% of males knew that drinking unboiled milk can cause tuberculosis. Very small proportion of participants knew major symptoms of a person with tuberculosis: “night sweating” (3.7% of females and 4.16% of males), “chest pain” (6.16% of females and 5.57% of males), and “fever” (9.16% of females and 9.99% of males) (Table 2). The most commonly identified TB symptom is “persistent cough” (66.6% of females and 77.02 of males, overall: 71.38%). The majority of respondents (84.38%) knew that “TB can be cured.” And 76.47% said they will not “keep TB patients in family a secret.”

### 3.3. Magnitude of Low TB Knowledge

The overall prevalence of low TB knowledge is high, with higher proportion among women, 44.05% (95% CI: 42.05–46.24%), compared to men, 32.3% (95% CI: 30.34–34.32%). Rural residents (49.17% of females and 35.44% of males) have higher prevalence of low TB knowledge compared with urban residents (28.1% among females and 21.11% among males). For both female and male respondents, the magnitude of low TB knowledge is higher among the youths (15–24 years) (46.17%) compared with older age groups. Respondents from both hard to reach and accessible regions have similar level of low TB knowledge (Table 3).

### 3.4. Socioeconomic Factors Associated with Low TB Knowledge

#### 3.4.1. Bivariate Analysis Results

At the bivariate analysis stage, the crude ORs showed that all the socioeconomic variables were significantly associated with low TB knowledge among both female and male study participants. After adjusting, all factors remained significant for females but residence, region, and occupation were not any more significantly associated with low TB knowledge in males (Tables 4 and 5).

#### 3.4.2. Multivariate Analysis Results for Female Study Participants

Rural women are victims of having low TB knowledge. The odds of low TB knowledge was 1.22 (95% CI: 1.06–1.41, *p* = 0.006) times higher among rural female residents as compared to urban residents. Being in the age groups 25–34, 35–44, and 45–49 was negatively associated with low TB knowledge compare with 15–24 years females, AORs being 0.68 (95% CI: 0.62–0.75), 0.60 (95% CI: 0.54–0.67), and 0.62 (95% CI: 0.53–0.71), respectively, and *p* < 0.0001 in all. Not having any formal education (AOR = 3.28, 95% CI: 2.52–4.27, *p* < 0.0001) and having attended only primary education (AOR = 1.95, 95% CI: 1.51–2.51, *p* < 0.0001) had increased odds of low TB knowledge as compared to women participants who have attended more than secondary school. The odds of low TB knowledge were higher among participants with lowest wealth quintiles, second, middle, and fourth wealth quintiles, as compared to those participants from highest wealth quartiles, AORs = 1.4 (95% CI: 1.20–1.63, *p* < 0.0001), 1.25 (95% CI: 1.07, 1.46, *p* = 0.005), 1.23 (95% CI: 1.06–1.44, *p* = 0.008), and 1.17 (95% CI: 1.01–1.35, *p* = 0.035), respectively. “Unskilled female manual workers,” being “agricultural employee,” those “not working,” “skilled manual” worker, and “clerical/sales and service workers” were associated with increased odds of low TB knowledge compared with professional/technical/managerial female workers, AORs being 4.15 (95% CI: 2.37–7.28, *p* < 0.0001), 2.28 (95% CI: 1.44–3.60, *p* < 0.001), 1.96 (95% CI: 1.25–3.08, *p* = 0.004), 1.81 (95% CI: 1.13–2.89, *p* = 0.013), and 1.70 (95% CI: 1.08–2.67, 0.023), respectively. Female participants who had no access to newspaper, television, or radio had 1.52 (95% CI: 1.41–1.64, *p* ≤ 0.0001) times increasing odds of low TB knowledge as compared to those who had access to either of the three media at least once a week (Tables 4 and 5).

#### 3.4.3. Multivariate Analysis Results for Male Study Participants

Not having any formal education and having attended primary education and secondary education had increased odds of having low TB knowledge as compared to male participants who have attended more than secondary school, AORs being 7.42 (95% CI: 5.70–9.66, *p* < 0.0001), 3.49 (95% CI: 2.71–4.48, *p* < 0.0001), and 1.55 (95% CI: 1.17–2.05, *p* = 0.002), respectively. The odds of low TB knowledge were higher among participants with lowest and middle wealth quintiles as compared to those participants from highest wealth quintile, AORs being 1.28 (95% CI: 1.11–1.48, *p* = 0.001) and 1.19 (95% CI: 1.03–1.36, *p* = 0.015), respectively. Male participants who had no access to newspaper, television, or radio had 1.71 (95% CI: 1.54–1.90, *p* < 0.0001) times higher odds of low TB knowledge as compared to those who had access to either of the three media at least once a week. The adjusted ORs for residence, occupation, and region did not show association in men and were excluded by the forward regression (Tables 6 and 7).

## 4. Discussion

In this study, though awareness of tuberculosis is high, specific knowledge on TB transmissions and symptoms is very low. Some major misconceptions on TB transmission are identified. The prevalence of low TB knowledge is high in the general adult population of Ethiopia, with higher magnitude among females than among males.

Rural women, younger people, less educated people, people from low socioeconomic status, those who have no access to mass media, and those who never heard of community conversation program on health and related topics have higher odds of low TB knowledge.

The level of low TB knowledge in the population in this study is in line with other small scale similar studies in different parts of Ethiopia [9, 14, 21, 22] and with studies done in other African countries and Asia [15, 16, 23, 30]. The high prevalence of low TB knowledge in the general population of Ethiopia might indicate that the current information and health education on TB is not sufficient and it is not properly addressing the target population. The situation is also exacerbated by the fact that majority of the population in Ethiopia live in low socioeconomic status and majority are from rural area (86.8%), with high rate of no formal education or only primary education (41.6% and 40.9%), no formal work (30.3%) or being only agricultural employees (38.3%), with lowest wealth index (21.4%), and no regular access to media (26.4%), or having not heard of community conversation (58.7%), which in turn are associated with low level of TB knowledge. As shown in the study by Gele et al. [18] and another study by Demissie et al. [17], low knowledge on TB is significantly associated with patient delay to seek health care, which is one of the major obstacles for controlling TB.

The finding regarding high misconception on TB transmission is comparable with other similar studies done in Africa and Asia [16, 31]. Understanding the misconceptions is very important as it might be the indication of the presence for delay of proper health seeking and refusal to take the right treatment decision and adherence to treatment. Misconception also may lead to stigma, which creates difficulty in engaging the community in TB control programs [11, 13]. Health education programs on TB need to address the common misconceptions in the community.

The fact that the rural women are highly affected with low TB knowledge compared to urban women could be due to the fact that the majority of rural women in Ethiopia are housewives confined to household activities with fewer outside social interaction compared to male in the society. They are also exposed to different economic, cultural, geographical barriers to get access to proper information. Similar findings from North Ethiopia were reported by Mesfin [21] and other study from Vietnam [32]. Addressing the rural women will contribute to the success of the TB control program.

The poor (low wealth quintile) have high prevalence of low comprehensive TB knowledge and it is in line with the study from India [16]. As proved by the study from Manila [15], people with low income have association with low health seeking behavior. The low level of knowledge among the poor in our study worsens the low level of health seeking behavior among the poor [17]. The burden of TB is high among the poor with deprived living conditions, malnutrition, and exposure to other communicable diseases. The lack of knowledge on TB in this community will aggravate the situation as they will not know how to protect themselves from the disease, when to seek health care, the need for treatment, and the importance of adherence to treatment for TB. As a result, these would in turn create difficulty to have effective TB control program. The poor need to be addressed in the health education and information on TB and addressing the problems of the poor will contribute to the effectiveness of TB control programs.

Education is highly associated with the level of comprehensive TB knowledge, and the uneducated people and those who had only primary education had high odds of low comprehensive knowledge. This finding was supported by similar other studies [9, 15, 21, 23, 33]. The explanation might be that the educated people have more access to different sources of information and easily understand more complex messages. Increasing the level of education in the community will increase the general knowledge about infection control including TB and the general health of the people.

Access to mass media is an important factor associated with TB knowledge. Those study participants who have access to either newspaper, TV, or radios at least once a week had better TB knowledge. Increasing the access to mass media and community health education in TB control through mass media can play a key role in creating awareness and building knowledge on TB. This may encourage individuals to seek health care on timely fashion, to adhere to treatment to protect them from TB which will contribute in all over reduction of prevalence of TB.

The secondary data analysis employed in this study could have been influenced by any unknown limitations related to the primary data method of data collection and sampling procedures.

Some variables which may have effect on TB knowledge and at the same time which were not collected in the primary data are not analysed in the current study. There might also be unmeasured confounding factors affecting TB knowledge as primary data was collected for a different purpose. The TB knowledge questions collected in the primary data were not complete. Information such as knowledge on TB prevention methods and importance of adherence to treatment could have been included.

Moreover, the composite index of low TB knowledge was taken from ten variables after doing factor analysis for consistency. Due to the small number of variables included in the index construction, the Chronbach Alpha was relatively in the lower border. Including more TB prevention knowledge items such as ventilation of the households, avoiding overcrowding, avoiding of spiting in all places, protecting people from directly coughing and sneezing, and importance of using mask for contacts with infectious TB patients may increase the reliability of the overall index.

## 5. Conclusion and Recommendation

The result from this study produced useful information for policy makers to evaluate and shape the TB control programs and plan socioeconomic and public health measures to increase TB knowledge and further enhance the TB control programs.

Public health measures should thus focus on enhancing the health education to address the most affected population group and increase the level of TB knowledge among the general population. The education materials need to be target oriented and incorporate the basic knowledge gaps such as concepts on TB transmission, TB major symptoms, curability, prevention methods, and the importance of treatment, at the same time of addressing the misconceptions. Increasing the use of multiple mass media sources would help to improve the health education coverage and address the target populations. Increasing the regular education coverage to the population and incorporating TB to school curriculum would especially address the uneducated and the in-school youths. Most importantly, tuberculosis control programs and intervention prioritizations should consider appropriate strategies for tuberculosis education, promotion, communication, and social mobilization to address the rural women, the youth, the poor, less educated people, and unskilled workers. Additionally, the depth of the magnitude of the problem needs to be addressed by socioeconomic measures such as empowering rural women to make their own decision and to get access to information and reformulating poverty reduction strategies to improve the socioeconomic status of the people.

Further large scale study is recommended to address the unmeasured confounders and additional TB knowledge questions.

## Figures and Tables

**Table 1:** Background characteristics of respondents, Ethiopia 2011.

Characteristics	Category	Number	Percentage
Gender	Female	**16,515**	**53.9**
	Male	**14,110**	**46.1**

Residence	Urban	9,545	31.2
	Rural	21,080	68.8

Age	15–24	12,019	40.9
	25–34	9,241	31.5
	35–44	6,061	20.6
	45–49	2,062	7.0

Current marital status	Never in union	10,066	32.9
	Married	17,017	55.6
	Living with partner	1,117	3.7
	Widowed	679	2.2
	Divorced	1,220	4.0
	No longer living together/separated	526	1.7

Region	Accessible regions	15,092	49.3
	Hard to reach regions	8,388	27.4
	City administrations and Harari region	7,145	23.3

Education	No education	12,727	41.6
	Primary	12,529	40.9
	Secondary	3,021	9.9
	More than secondary	2,348	7.7

Wealth quintile	Lowest	6,558	21.4
	Second	4,511	14.7
	Middle	4,422	14.4
	Fourth	4,909	16.0
	Highest	10,225	33.4

Occupation	Not working	9,183	30.3
	Professional/technical/managerial	1,199	4.0
	Clerical/sales/services	5,517	18.2
	Agricultural employee	11,608	38.3
	Skilled manual	2,431	8.0
	Unskilled manual	404	1.3

Exposures to mass media	Accesses either newspaper, TV, or radio less than or at least once a week	22,455	73.6
	Access none of the three media	8,065	26.4

Heard of community conversation program	No	17,959	58.7
	Yes	12,652	41.3

Last time attended community conversation meeting	Never attended	2,977	23.6
	Within last 3 months	3,598	28.5
	4–11 months ago	1,478	11.7
	One year or more ago	1,657	13.1

**Table 2:** TB awareness and respondents correct knowledge on TB transmission, symptoms, and treatment, Ethiopia 2011.

Characteristics	Characteristics (correct response)	Women *n* (%)	Men *n* (%)	Total *n* (%)
Awareness	Heard of tuberculosis or TB (yes)	15,045 (89.92)	13,518 (95.09)	28,563 (92.3)

Transmission: tuberculosis spread by	Air when coughing or sneezing (yes)	8,644 (49.97)	9,036 (61.84)	17,680 (55.44)
	Sharing utensils (no)	11,568 (71.6)	8,673 (65.33)	20,241 (68.71)
	Touching a person with TB (no)	14,864 (92.32)	12,587 (90.74)	27,451 (91.59)
	Food (no)	15,284 (93.5)	13,093 (93.03)	28,377 (93.29)
	Sexual contact (no)	15,656 (95.94)	13,513 (96.97)	29,169 (96.42)
	Mosquito bites (no)	16,379 (99.47)	13,958 (99.19)	30,337 (99.34)
	Drinking unboiled milk (yes)	312 (1.82)	423 (2.55)	735 (2.16)
	Exposure to cold (no)	12,894 (79.22)	11,124 (80.12)	24,018 (79.64)

Symptom/signs: symptoms of a person with TB	Persistent cough (>2 weeks) (yes)	11,354 (66.56)	11,141 (77.02)	22,495 (71.38)
	Weight loss (yes)	5,250 (28.44)	6,131 (42.54)	11,381 (34.94)
	Poor appetite (yes)	1,870 (9.304)	1,940 (15.15)	3,810 (12)
	Night sweating (yes)	905 (3.7)	853 (4.16)	1,758 (3.91)
	Chest pain (yes)	1,033 (6.16)	1,020 (5.57)	2,053 (5.89)
	Fever (yes)	2,121 (9.16)	1,974 (9.99)	4,095 (9.54)

Treatment	Tuberculosis can be cured (yes)	12,564 (79.8)	12,302 (89.45)	24,866 (84.38)

Attitude	Will keep TB patient in family a secret (no)	10,724 (72.12)	11,025 (81.25)	21,749 (76.47)

**Table 3:** Prevalence of low comprehensive TB Knowledge, Ethiopia 2011.

Characteristics	Category	Women		Men	
		Prevalence	95% CI	Prevalence	95% CI
National total		44.14	(42.05, 46.24)	32.3	(30.34, 34.32)

Residence	Urban	28.1	(24.55, 31.95)	21.11	(18.29, 24.24)
	Rural	49.17	(46.69, 51.66)	35.44	(33.06, 37.9)

Age	15–24	46.31	(43.74, 48.9)	39.92	(37.18, 42.74)
	25–34	42.57	(39.72, 45.47)	27.6	(25.24, 30.08)
	35–44	41.5	(38.37, 44.71)	27.19	(24.61, 29.93)
	45–49	45.36	(41.02, 49.77)	27.42	(23.27, 32.0)

Region	Accessible regions	45.4	(43.07, 47.75)	33.14	(30.97, 35.37)
	Hard to reach regions	41.59	(8.09, 45.18)	34.31	(31.03, 37.75)
	City administrations	27.51	(24.79, 30.42)	17.95	(14.34, 22.23)

Current marital status	Never in union	42.11	(39.4, 44.87)	36.87	(34.24, 39.57)
	Married	44.66	(42.03, 47.32)	28.79	(26.66, 31.02)
	Living with partner	42.96	(37.22, 48.9)	31.87	(24.1, 40.8)
	Widowed	39.84	(34.23, 45.73)	37.95	(22.82, 55.84)
	Divorced	52.83	(47.14, 58.45)	41.79	(33.78, 50.27)
	No longer living together/separated	42.72	(35.54, 50.21)	22.6	(12.84, 36.65)

Education	No education	52.67	(50.01, 55.32)	44.01	(40.85, 47.21)
	Primary	40.09	(37.68, 42.56)	31.16	(28.84, 33.58)
	Secondary	22.41	(18.72, 26.59)	15.8	(13.16, 18.85)
	More than secondary	13.99	(10.36, 18.61)	8.251	(6.038, 11.18)

Wealth quintile	Lowest	55.31	(51.78, 58.8)	42.7	(38.29, 47.22)
	Second	50.77	(47, 54.53)	37.05	(33.63, 40.61)
	Middle	49.63	(45.9, 53.36)	35.66	(32.09, 39.39)
	Fourth	43.69	(40.33, 47.11)	30.15	(26.9, 33.62)
	Highest	27.93	(24.86, 31.23)	20.67	(17.8, 23.87)

Occupation	Professional/technical/managerial	9.023	(5.007, 15.73)	7.766	(5.317, 11.21)
	Not working	45.25	(42.51, 48.01)	34.94	(30.09, 40.12)
	Clerical/sales and services	36.02	(33.35, 38.78)	24.66	(20.87, 28.89)
	Agricultural employee	51.38	(48.06, 54.7)	35.84	(33.4, 38.35)
	Skilled manual	41.52	(35.84, 47.44)	21.01	(17.13, 25.5)
	Unskilled manual	55.6	(47.14, 63.74)	29.98	(20.37, 41.73)

Exposure to mass media	Accesses none of the three media at least once a week	57.17	(54.52, 59.77)	50.75	(46.78, 54.72)
	Accesses either newspaper, TV, or radio at least once a week	37.87	(35.66, 40.14)	28.31	(26.41, 30.3)

Heard of community conversation program	No	50.1	(47.62, 52.58)	42.1	(39.45, 44.79)
	Yes	30.71	(28.51, 32.99)	23.35	(21.24, 25.59)

**Table 4:** Factors associated with low TB knowledge among women, Ethiopia 2011.

Characteristics	Category	Low knowledge *n* (%)	Crude OR (95% CI)	*p* value (crude)	Adjusted OR (95% CI)	*p* value (adjusted)
Residence	Urban	1,344 (28.1)	1		1	
	Rural	5,253 (49.17)	2.48 (2.01, 3.05)	<0.0001	1.22 (1.06, 1.41)	0.006

Age	15–24	2,907 (46.31)	1		1	
	25–34	2,057 (42.57)	0.86 (0.76, 0.97)	0.016	0.68 (0.62, 0.75)	<0.0001
	35–44	1,212 (41.5)	0.82 (0.71, 0.95)	0.010	0.60 (0.54, 0.67)	<0.0001
	45–49	421 (45.36)	0.96 (0.80, 1.16)	0.688	0.62 (0.53, 0.71)	<0.0001

Current marital status	Never in union	1,673 (42.11)	1		1	
	Married	3,897 (44.66)	1.11 (0.96, 1.28)	0.152	0.82 (0.74, 0.91)	<0.0001
	Living with partner	275 (42.96)	1.04 (0.80, 1.34)	0.787	0.92 (0.77, 1.10)	0.362
	Widowed	216 (39.84)	0.91 (0.71, 1.17)	0.465	0.75 (0.61, 0.93)	0.009
	Divorced	400 (52.83)	1.54 (1.21, 1.96)	<0.0001	1.22 (1.04, 1.44)	0.017
	No longer living together/separated	136 (44.14)	1.03 (0.75, 1.40)	0.876	1.03 (0.81, 1.32)	0.807

Region	Accessible regions	3,578 (45.4)	2.19 (1.85, 2.60)	<0.0001	0.91 (0.77, 1.08)	0.293
	Hard to reach regions	2,013 (41.59)	1.88 (1.53, 2.30)	<0.0001	0.68 (0.54, 0.85)	0.001
	City administrations	1,006 (27.51)	1		1	

Education	No education	4,013 (52.67)	6.84 (4.80, 9.76)	<0.0001	3.28 (2.52, 4.27)	<0.0001
	Primary	2,227 (40.09)	4.12 (2.94, 5.76)	<0.0001	1.95 (1.51, 2.51)	<0.0001
	Secondary	260 (22.41)	1.78 (1.23, 2.56)	0.002	1.17 (0.89, 1.55)	0.261
	More than secondary	97 (13.99)	1		1	

Wealth quintile	Lowest	1,873 (55.31)	3.19 (2.59, 3.94)	<0.0001	1.40 (1.20, 1.63)	<0.0001
	Second	1,162 (50.77)	2.66 (2.14, 3.31)	<0.0001	1.25 (1.07, 1.46)	0.005
	Middle	1,094 (49.63)	2.541 (2.05, 3.15)	<0.0001	1.23 (1.06, 1.44)	0.008
	Fourth	1,058 (43.69)	2.00 (1.66, 2.42)	<0.0001	1.17 (1.01, 1.35)	0.035
	Highest	1,410 (27.93)	1		1	

**Table 5:** Factors associated with low TB knowledge among women, Ethiopia 2011, continued.

Characteristics	Category	Low knowledge *n* (%)	Crude OR (95% CI)	*p* value (crude)	Adjusted OR (95% CI)	*p* value (adjusted)
Occupation	Professional/technical/managerial	27 (9.02)	1		1	
	Not working	3,292 (45.25)	8.33 (4.45, 15.58)	<0.0001	1.96 (1.25, 3.09)	0.004
	Clerical/sales and services	1,138 (36.02)	5.68 (3.01, 10.69)	<0.0001	1.70 (1.08, 2.67)	0.023
	Agricultural employee	1,531 (51.38)	10.66 (5.58, 20.35)	<0.0001	2.28 (1.44, 3.60)	<0.0001
	Skilled manual	490 (41.52)	7.16 (3.63, 14.14)	<0.0001	1.81 (1.13, 2.89)	0.013
	Unskilled manual	57 (55.6)	12.62 (6.14, 25.96)	<0.0001	4.15 (2.37, 7.28)	<0.0001

Exposure to mass media	Accesses none of the three media at least once a week	2,987 (57.17)	2.19 (1.96, 2.44)	<0.0001	1.52 (1.41, 1.64)	<0.0001
	Accesses either newspaper, TV, or radio at least once a week	3,585 (37.87)	1		1	

Heard of community conversation program	No	5,203 (50.1)	2.27 (1.99, 2.57)	<0.0001	1.63 (1.52, 1.76)	<0.0001
	Yes	1,385 (30.71)	1		1	

Final model fit	Pseudo-*R* ^2^ = 0.08, Log Likelihood = −10360.78, LR *χ* ^2^ (25) = 1680.62, *p* < 0.0001					

**Table 6:** Factors associated with low TB knowledge among men, Ethiopia 2011.

Characteristics	Category	Low knowledge *n* (%)	Crude OR (95% CI)	*p* value (crude)	Adjusted OR (95% CI)	*p* value (adjusted)
Residence	Urban	664 (21.11)	1			
	Rural	3,477 (35.44)	2.05 (1.67, 2.53)	<0.0001		

Age	15–24	1,871 (39.92)	1		1	
	25–34	1,010 (27.6)	0.57 (0.51, 0.65)	<0.0001	0.59 (0.52, 0.68)	<0.0001
	35–44	679 (27.19)	0.56 (0.49, 0.65)	<0.0001	0.54 (0.46, 0.63)	<0.0001
	45–49	244 (27.42)	0.57 (0.46, 0.71)	<0.0001	0.49 (0.40, 0.60)	<0.0001

Current marital status	Never in union	1,833 (36.87)	1		1	
	Married	2,061 (28.79)	0.69 (0.62, 0.78)	<0.0001	0.73 (0.64, 0.84)	<0.0001
	Living with partner	100 (31.87)	0.80 (0.54, 1.20)	0.280	1.17 (0.87, 1.57)	0.307
	Widowed	22 (37.95)	1.05 (0.51, 2.17)	0.901	0.74 (0.35, 1.60)	0.447
	Divorced	99 (41.79)	1.23 (0.87, 1.74)	0.245	1.14 (0.84, 1.54)	0.400
	No longer living together/separated	26 (22.6)	0.50 (0.25, 1.00)	0.050	0.57 (0.34, 0.96)	0.033

Region	Accessible regions	2,410 (33.14)	2.27 (1.70, 3.01)	<0.0001		
	Hard to reach regions	1,319 (34.31)	2.39 (1.76, 3.24)	<0.0001		
	City administrations	412 (17.95)	1	1		

Education	No education	1,834 (44.01)	8.74 (6.14, 12.44)	<0.0001	7.42 (5.70, 9.66)	<0.0001
	Primary	1,989 (31.16)	5.03 (3.54, 7.16)	<0.0001	3.49 (2.71, 4.48)	<0.0001
	Secondary	224 (15.8)	2.09 (1.39, 3.13)	<0.0001	1.55 (1.17, 2.05)	0.002
	More than secondary	94 (8.251)	1		1	

Wealth quintile	Lowest	1,144 (42.7)	2.86 (2.21, 3.70)	<0.0001	1.28 (1.11, 1.48)	0.001
	Second	785 (37.05)	2.26 (1.78, 2.87)	<0.0001	1.06 (0.92, 1.22)	0.390
	Middle	768 (35.66)	2.13 (1.67, 2.71)	<0.0001	1.19 (1.03, 1.36)	0.015
	Fourth	723 (30.15)	1.66 (1.31, 2.09)	<0.0001	1.01 (0.89, 1.16)	0.828
	Highest	721 (20.67)	1		1	

**Table 7:** Factors associated with low TB knowledge among Men, Ethiopia 2011, continued.

Characteristics	Category	Low knowledge *n* (%)	Crude OR (95% CI)	*p* value (crude)	Adjusted OR (95% CI)	*p* value (adjusted)
Occupation	Professional/technical/managerial	63 (7.77)	1			
	Not working	340 (34.94)	6.38 (4.03, 10.08)	<0.0001		
	Clerical/sales and services	403 (24.66)	3.89 (2.44, 6.19)	<0.0001		
	Agricultural employee	3,030 (35.84)	6.63 (4.42, 9.96)	<0.0001		
	Skilled manual	201 (21.01)	3.16 (1.99, 5.02)	<0.0001		
	Unskilled manual	64 (29.98)	5.08 (2.69, 9.60)	<0.0001		

Exposure to mass media	Accesses none of the three media at least once a week	1,096 (50.75)	2.61 (2.23, 3.05)	<0.0001	1.71 (1.54, 1.90)	<0.0001
	Accesses either newspaper, TV, or radio at least once a week	3,027 (28.31)	1		1	

Heard of community conversation program	No	5,203 (42.1)	2.39 (2.10, 2.71)	<0.0001	1.71 (1.57, 1.85)	<0.0001
	Yes	1,385 (23.35)	1		1	

Final model fit	Pseudo-*R* ^2^ = 0.104, Log Likelihood = −7164.00, LR *χ* ^2^ (17) = 1655.86, *p* < 0.0001					
